# Activation of NF-kappa B Signaling Promotes Growth of Prostate Cancer Cells in Bone

**DOI:** 10.1371/journal.pone.0060983

**Published:** 2013-04-05

**Authors:** Renjie Jin, Julie A. Sterling, James R. Edwards, David J. DeGraff, Changki Lee, Serk In Park, Robert J. Matusik

**Affiliations:** 1 Vanderbilt Prostate Cancer Center and Department of Urologic Surgery, Vanderbilt University Medical Center, Nashville, Tennessee, United States of America; 2 Vanderbilt Center for Bone Biology, Vanderbilt University Medical Center, Nashville, Tennessee, United States of America; 3 Department of Veterans Affairs, Tennessee Valley Healthcare System (Nashville), Nashville, Tennessee, United States of America; 4 Nuffield Department of Orthopaedics, Rheumatology and Musculoskeletal Sciences, University of Oxford, Oxford, United Kingdom; University of Pittsburgh School of Medicine, United States of America

## Abstract

Patients with advanced prostate cancer almost invariably develop osseous metastasis. Although many studies indicate that the activation of NF-κB signaling appears to be correlated with advanced cancer and promotes tumor metastasis by influencing tumor cell migration and angiogenesis, the influence of altered NF-κB signaling in prostate cancer cells within boney metastatic lesions is not clearly understood. While C4-2B and PC3 prostate cancer cells grow well in the bone, LNCaP cells are difficult to grow in murine bone following intraskeletal injection. Our studies show that when compared to LNCaP, NF-κB activity is significantly higher in C4-2B and PC3, and that the activation of NF-κB signaling in prostate cancer cells resulted in the increased expression of the osteoclast inducing genes PTHrP and RANKL. Further, conditioned medium derived from NF-κB activated LNCaP cells induce osteoclast differentiation. In addition, inactivation of NF-κB signaling in prostate cancer cells inhibited tumor formation in the bone, both in the osteolytic PC3 and osteoblastic/osteoclastic mixed C4-2B cells; while the activation of NF-κB signaling in LNCaP cells promoted tumor establishment and proliferation in the bone. The activation of NF-κB in LNCaP cells resulted in the formation of an osteoblastic/osteoclastic mixed tumor with increased osteoclasts surrounding the new formed bone, similar to metastases commonly seen in patients with prostate cancer. These results indicate that osteoclastic reaction is required even in the osteoblastic cancer cells and the activation of NF-κB signaling in prostate cancer cells increases osteoclastogenesis by up-regulating osteoclastogenic genes, thereby contributing to bone metastatic formation.

## Introduction

Almost all patients with advanced prostate cancer (PCa) develop osseus metastasis. The development of tumor growth in the bone is the most critical complication of advanced PCa, frequently resulting in significant morbidity and mortality [1]. Unlike other types of cancer, an initial metastatic deposit of PCa cells is almost strictly limited to bone and is often the only site of distal spread even in late stages of disease [2]. Once prostate tumor cells enter the skeleton, a destructive cycle of gross skeletal damage and tumor growth occurs, at which point curative therapy is no longer possible and palliative treatment becomes the only option. The median time between the diagnosis of a clinically evident skeletal metastasis and death is approximately 3–5 years [3]. Therefore, understanding the mechanism by which the PCa cells thrive within the bone environment and developing effective method(s) to prevent or treat PCa bone metastasis is critical to increase the survival rate of advanced PCa patients.

Unlike other solid tumors that are associated with osteolytic bone metastases, PCa bone metastasis is associated with osteoblastic metastasis. However, the successful colonization of the bone by PCa cells requires both osteolytic and osteoblastic processes. This occurs in part because PCa cells are capable of producing growth factors that can affect both osteoblasts and osteoclasts, resulting in osteoblastic bone formation and excessive bone resorption [1], [4]. While the role of osteoblasts in PCa bone metastasis is well recognized, several findings strongly suggest an important role for osteoclast function in the successful formation of PCa bone metastases [5]–[10]. For example, when PCa cells initially colonize a bone, they are thought to first induce osteoclastogenesis [11], and subsequent bone resorption. Histomorphometric evidence indicates that osteoblastic metastases form in trabecular bone at sites of previous osteoclast resorption and such resorption is required for subsequent osteoblastic bone formation [12]. These findings suggest that PCa induces bone deposition through an overall increase in bone remodeling. Additionally, osteoclastic bone resorption contributes to the majority of skeletal sequelae, or skeletal-related events (SREs, such as fracture and pain), in patients with bone metastases. Further, osteoclastic bone resorption also contributes to the establishment of tumors in the skeleton. Therefore, osteoclastogenesis induced by PCa cells is suggested to be an early event of bone metastasis and is a necessary initial prerequisite for PCa bone colonization. Although the concept of osteoclast activation as an underlying component of PCa growth in bone is already well recognized, the mechanistic details by which the PCa cells increase osteoclast activation and subsequently induce metastasis to the bone environment are still unclear.

It is now widely believed that the molecular triad - Receptor Activator of NF-κB Ligand (RANKL), its receptor RANK, and the endogenous soluble RANKL inhibitor, osteoprotegerin (OPG) - play essential and direct roles in the formation, function, and survival of osteoclasts. Many studies have indicated that RANKL/RANK/OPG are the key regulators of bone metabolism both in normal and pathological conditions, including PCa bone metastases [13], [14]. Another important gene, Parathyroid hormone-related protein (PTHrP), is known to be involved to osteoclast differentiation. PTHrP is produced by virtually all tumors that metastasize to the bone, and numerous studies have demonstrated a correlation between PTHrP expression and skeletal localization of tumors. PTHrP has prominent effects in bone via its interaction with the PTH-1 receptor on osteoblastic cells. Through indirect means, PTHrP supports osteoclastogenesis by up-regulating RANKL in osteoblasts [15]. PCa cells have been shown to express several factors that regulate osteoclastogenesis, including PTHrP, macrophage colony-stimulating factor (M-CSF), members of the transforming growth factor β (TGF-β) superfamily, and urokinase-type plasminogen activator (uPA-plasmin), resulting in the activation of the matrix metalloproteinases (MMPs; specifically MMP-2 and MMP-9) as well as interleukin-1 (IL-1) and interleukin-6 (IL-6) [16]. However, identification of the critical mechanism(s) or primary pathway(s) that responsible for the initial expression of osteoclastogenic gene(s) and excessive resorption resulting in the promotion of PCa bone metastasis remains elusive.

It is widely accepted that RANKL/RANK/OPG are the key regulators of bone metabolism and the PTHrP is one of the most important key regulators in osteoclast and osteoblast differentiation. Therefore, in this present study we have focused on understanding how RANKL and PTHrP are regulated in PCa cells by NF-κB. NF-κB proteins are an important class of transcriptional regulators in PCa. Abundant data supports a key role for the NF-κB signaling pathway in controlling the initiation and progression of human cancer [17]–[20]. The over-expression of NF-κB in the nucleus of PCa cells appears to be correlated with PCa chemoresistance, advanced stage, PSA recurrence and metastatic spread [21]–[27]. Several studies have published evidence indicating the NF-κB pathway is a contributor to visceral or “soft-tissue” metastasis in PCa [18], [19], [28], [29]. Previously, we have reported that activation of NF-κB signaling promotes castrate-resistant growth of PCa [30]. In this study, we investigated the role of NF-κB signaling in the colonization of PCa cells within the bone environment and the role of this mechanism plays in the skeletal destruction associated with PCa bone metastasis. We report that the activation of NF-κB signaling increases the expression of osteoclastogenic genes in PCa cells, which is sufficient for cancer cells to attach and grow in the bone environment and enhance lesion formation. To our knowledge, this is the first report that activation of NF-κB signaling in PCa cells increases osteoclastogenesis by up-regulating osteoclastogenic genes, thereby contributing to bone metastases formation. Our study indicates that targeting down-regulation of the NF-κB could have a major impact on reducing painful bone metastasis in advanced PCa patients.

## Materials and Methods

### Cell culture and materials

The human prostate carcinoma cell lines LNCaP and PC-3 were obtained from ATCC (Manassas, VA). C4-2B cells were gifts of Dr. Leland Chung (Cedars Sinai Medical Center, Los Angeles, CA) [31]. Cells were maintained at 37°C in a humidified atmosphere of 5% CO_2_ in the air. Cell lines were routinely cultured in RPMI 1640 (Gibco-BRL) medium containing 5% fetal calf serum (FBS) (Hyclone), 0.1% ITS and 0.1% Glutamine (Gibco-BRL).

### Generation of NF-κB signaling continuously activated/inactivated PCa cell lines

To generate a constitutively activated NF-κB signaling PCa cell line, LNCaP cells were stably infected with IKK2-EE retroviral vector resulting in LNCaP-EE, in which NF-κB activity was activated with a constitutively active (EE) mutants of IKK2 [32], [33]. To generate NF-κB signaling continuously inactivated PCa cell lines, C4-2B and PC3 cells were stably infected with IKK2-KD retroviral vector (C4-2B-KD and PC3-KD), in which NF-κB activity was inhibited with a kinase dead (KD) IKK2 mutant [32], [33]. The cells infected with empty vector were used as controls (LNCaP-EV, C4-2B-EV and PC3-EV). All retroviral vectors were a gift from Dr. Martin Leverkus, University of Magdeburg, Germany.

### Reverse Transcription and Real-time PCR

Total RNAs from LNCaP, LNCaP-EE, C4-2B and PC3 cells were extracted using Trizol (Gibco-BRL), and residual genomic DNA was removed by DNaseI (Invitrogen) treatment. The RNAs were reverse transcribed using random primers and Superscript II (Gibco-BRL) according to the manufacturer's protocol. The primers used to amplify RANKL were 5′-TGGAAGGCTCATGGTTGGAT-3′ (forward), 5′-CATTGATGGTGAGGTGTGCAA-3′ (reverse); PTHrP were 5′-TTAAAGCAGTACCCCCCTACCA-3′ (forward), 5′-ATGGGCTCTAGCGCCTCTCT-3′ (reverse). Real-time PCR reactions were carried out in a 20 µl volume using a 96-well plate format and fluorescence was detected utilizing the Bio-Rad I-Cycler IQ Real-time detection system. Gene expression was normalized to 18s rRNA by the 2^−ΔΔCt^ method [34].

### RANKL ELISA and PTHrP Radio-immuno assays (RIA)

To determination of RANKL and PTHrP levels secreted by the PCa cells, LNCaP-EV, LNCaP-EE, PC3-EV and PC3-KD cells were cultured separately for 48 hours; cell numbers were counted. 40 and 100 µL of culture media were used in the ELISA (MyBioSource) and RIA (Beckman Coulter) per the manufacturer's instructions, respectively. Each sample was analyzed in triplicate by ELISA reader or a gamma counter (Beckman Coulter) and concentrations were determined per the manufacturer's instructions.

### Western blot analysis

Whole cell lysate was extracted from NF-κB activated/inactivated PCa cells. A 20 µg aliquot of each protein sample was separated on a 4 to 12% Tris-glycine gradient gel (NOVEX™), and then transferred to nitrocellulose membranes (Schleicher & Schuell, Germany). The membranes were blocked with 5% skim milk in TBS-T (Trypsin buffered saline, 1% Tween-20) buffer. The RANKL (N19, Santa Criuz) or PTHrP (N19, Santa Criuz) antibodies were added at their optimal concentration (1∶1000) and the blots were incubated 1 hour in room temperature. After washing three times for 10 minutes each in TBS-T, incubation was performed for 1 hour with the secondary horseradish-peroxidase-conjugated goat anti-mouse antibodies, respectively. The signals were developed by an ECL detection system (Amersham Biosciences, Amersham, USA).

### Transient transfection assay

The NGL vector [a NF-κB responsive reporter vector which has Luciferase and Green Fluorescent Protein (GFP) reporter genes] [35] was used to measure NF-κB activity in the PCa cancer cells by transient transfection experiments. LNCaP, C4-2B and PC3 cells were plated at an initial density of 2.5×10^4^/well in 24-well tissue culture plates. After 24 hours, the cells were transfected with NGL vector using Lipofectamine (Invitrogen) for four hours according to the manufacturer's protocol. The transfection efficiency was determined by co-transfecting pRL-CMV containing the Renilla luciferase reporter gene (Promega). Luciferase activity was determined using the Promega Corp luciferase assay system 24 hours after transfection. The values plotted represent the mean of at least three individual samples ± SEM.

### Osteoclast differentiation assay

Bone marrow cells were isolated from mouse femurs by fluid pressure applied with a syringe. To obtain bone marrow-derived monocyte/macrophages, the bone marrow cells were cultured in the DMEM medium containing 10% L929 supernatant as a source of M-CSF and RANKL [36]. After the primary cultured bone marrow cells differentiated into monocytes/macrophages (about 6 days), the cells were seeded in 24-well plate and treated with conditioned medium from different PCa cells (LNCaP, LNCaP-EE, C4-2B and PC3 cells). To generate the conditioned media containing secretions of PCa cells, RPMI 1640 medium (containing 5% FBS, 0.1% ITS and 0.1% Glutamine) was added to the PCa cell culture dish. After 24 hours, the medium was harvested and transferred to target cells (monocytes/macrophages). The condition medium was changed daily. Then, after 10 days of additional culture with conditioned media, the target cells were fixed and the osteoclast differentiation was confirmed by Tartrate-Resistant Acid Phosphate (TRAP) staining (Sigma). Cells with a positive staining for TRAP containing 3 or more nuclei were counted as osteoclasts. For quantification, the number of osteoclasts in each well of 24-well plate was counted under the microscope. Each group has 6 wells (wells of 24-well plate). The results showed as Mean Number ± Standard Deviations.

### Osteoblasts proliferation assay

One 3-day old wild-type C57BL6 pup was anesthetized on ice after dip in 70% ethanol. Then sacrificed by decapitation with scissors, the scalp was made a midline incision and exposed the cranium. Cranium (frontal, parietal, temporal and occipital bones) was excised with a pair of fine iris scissors and removed brain in α-MEM media (serum-free) on 60 mm dish. Cranial bone was cut into 4–5 pieces. Bones were put into 15 ml conical tube with 2 ml of collagenase media [serum-free α-MEM media with collagenase A (2 mg/ml)]. The bones were incubated for 30 minutes at 100 rpm in 37°C bacterial shaker. Supernatant media was gently discarded, and bones were added 5 ml of new collagenase media and incubated for 2 hrs at 150 rpm in shake incubator. After centrifuge at 1500 rpm for 3 min, supernatant media was gently discarded and re-suspended 5 ml in α-MEM supplemented with 10% FBS and 2× Penn/Strep and grew on 60 mm dish. After 2 weeks, the primary cultured osteoblasts were seeded in 96-well plats and treated with conditioned medium from NF-κB activated/inactivated PCa cells (LNCaP-EV, LNCaP-EE; C4-2B-EV, C4-2B-KD; PC3-EV and PC3-KD cells). To generate the conditioned media containing secretions of PCa cells, RPMI 1640 medium (containing 5% FBS, 0.1% ITS and 0.1% Glutamine) was added to the PCa cell culture dishes. After 24 hours, the medium was harvested and transferred to target cells (osteoblasts). MTT assay was performed at 48 hours after treatment.

### Mouse model of tumor-induced bone disease

All animal studies were carried out in strict accordance with the recommendations in the Guide for the Care and Use of Laboratory Animals of the National Institutes of Health. The protocol was approved by the Vanderbilt Institutional Animal Care & Use Committee (Permit Number: M/09/387). Both the NF-κB activated (PC3-EV, C4-2B-EV and LNCaP-EE) and inactivated (PC3-KD, C4-2B-KD and LNCaP-EV) PCa cells were used to investigate whether the activation of NF-κB signaling contributes to PCa tumor establishment and growth in the bone environment. To reduce inter-host variability when comparing control and test groups, NF-κB activated and inactivated PCa cells were grafted in the same mouse. In each individual host, the left tibia was injected with NF-κB activated PCa (PC3-EV, C4-2B-EV and LNCaP-EE) cells, whereas the right tibia was injected with NF-κB inactivated PCa (PC3-KD, C4-2B-KD and LNCaP-EV) cells, respectively. Specifically, a single-cell suspension of 2×10^5^ PC3-EV or PC3-KD; C4-2B-EV or C4-2B-KD; LNCaP-EV or LNCaP-EE cells/10 µl PBS was placed directly into the 6–7 week old male athymic nude mice (BALB/c strain) bone by intratibial injection under the isoflurane anesthesia, respectively. Each group had a minimum of 4 mice. Osteoblastic lesions were assessed using small animal X-ray radiograph imaging (Faxitron LX-60; Lincolnshire). Following sacrifice, bone volume was evaluated by micro CT scanning and histological analysis at 3 (for PC3-EV and PC3-KD cells injected mice) and 10 (for LNCaP-EV, LNCaP-EE, C4-2B-EV and C4-2B-KD cells injected mice) weeks after tumor inoculation. Harvested tibiae were fixed in 10% neutral buffered-formalin solution for 24 hours and decalcified in 0.5 mol/L EDTA in Ca^2+^- and Mg^2+^-free Dulbecco's PBS for one week before embedding in paraffin. Six-micrometer longitudinal serial sections were cut from the tibia and stained with H&E or by IHC to analyze tumor growth in the bone. Histomorphometric analyses for tumor volume (TV) and trabecular bone volume (BV) were performed using the Metamorph software (Molecular Devices, Inc.) as described previously [37].

### Microcomputed tomography (μCT) analysis

For the determination of the three-dimensional architecture of the bone following tumor inoculation, mice were sacrificed and the tibiae were collected and fixed overnight in 10% neutral buffered-formalin solution for 24 hours, followed by 70% ethanol. Samples were scanned using a μCT system (Scanco μCT 40; Bassersdorf, Switzerland). The scan was composed of 129 slices, with a threshold value of 263. A three-dimensional reconstruction of the images was done with the region of interest consisting of trabecular areas. Regions of interest were drawn on each of the 100 slices, just inside the cortical bone, to include only the trabecular bone and the marrow.

### Immunohistochemistry

Paraffin-embedded tissue sections of the tibiae were stained immunohistochemically with antibodies against AR (clone N20, Santa Cruz), PSA (clone LK2H10, Santa Cruz) and Fox A1 (clone T20, Santa Cruz). The primary antibody was incubated at the appropriate concentration (AR: 1∶1000; PSA: 1∶500; Fox A1: 1∶1000) for one hour at room temperature. The secondary antibody was incubated for 60 minutes, using either horseradish-peroxidase-conjugated goat anti-rabbit or goat anti-mouse (1∶1000), respectively. Slides were rinsed extensively in tap water, counterstained with Mayer's hematoxylin and mounted.

### Statistical and image analysis

Where appropriate, experimental groups were compared using two-sample *t*-test, with significance defined as *P*<0.05.

## Results

### Activation of NF-κB signaling increases expression of osteoclastogenesis-associated genes in PCa cells

**Figure 1:**
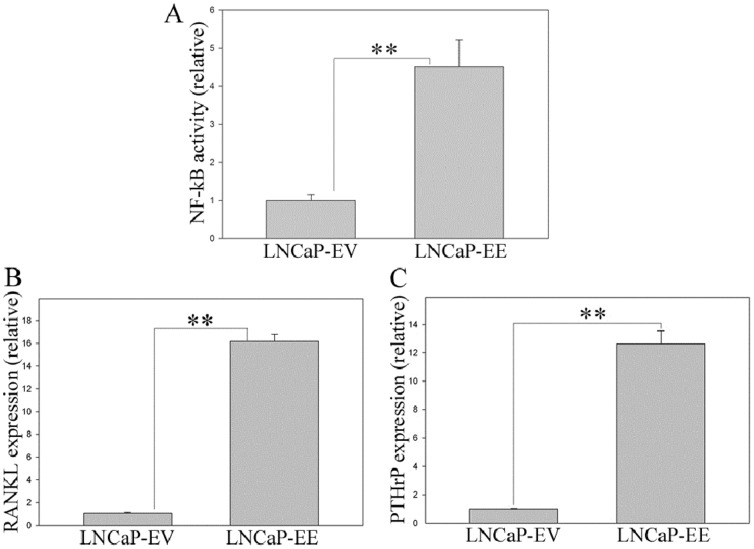
Activation of NF-κB signaling increases RANKL and PTHrP expression in PCa cells. NF-κB signaling was activated in LNCaP cells by infecting with IKK2-EE retroviral vector (LNCaP-EE), while LNCaP cells infected with the empty vector were used as the controls (LNCaP-EV). NF-κB activity in LNCaP-EV and LNCaP-EE cells were measured by using a NF-κB responsive NGL reporter (A). Expression of RANKL (B) and PTHrP (C) was measured by qRT-PCR. The values plotted represent the mean of at least three individual samples ± SEM. Statistical significance was determined by two-sample *t*-test. **, *P*<0.01.

IκBα inhibits NF-κB signaling by binding to NF-κB and preventing nuclear localization [38], [39]. Therefore, down regulation of IκBα results in continuous activation of NF-κB signaling [40], [41]. In order to investigate the role of NF-κB signaling on the prostate/PCa development and progression, we performed RNA microarray to directly compare prostate tissues from IκBα knock out (KO) mouse [40], [41] (including IκBα−/− and IκBα+/− mice) with normal mouse prostate tissues. The results from microarray analysis showed that the expression of many osteoclastogenesis-associated genes, such as RANKL, PTHrP, GM-CSF and uPA, increased over 2-fold in both IκBα−/− and IκBα+/− prostatic tissues, compared with that of normal prostate (data not shown). In order to further confirm whether NF-κB signaling is involved in the regulation of osteoclastogenesis-associated genes in PCa cells, we activated NF-κB signaling in LNCaP cells by infecting with IKK2-EE retroviral vector, in which NF-κB activity was activated with a constitutively active mutants of IKK2 [32], [33]. The activation of NF-κB signaling was confirmed using the NGL reporter, an NF-κB reporter plasmid which has an NF-κB responsive element coupled to a GFP/Luciferase reporter [35] (**Fig. 1A**). qRT-PCR analysis showed that RANKL and PTHrP expression are significantly increased in NF-κB activated LNCaP cells, compared to empty vector control cells (**Fig. 1B and 1C**). RANKL and PTHrP are known as key factors of osteoclastogenesis in the bone, and they play a key role in the bone metastasis of many cancers [15], [42]–[45]. Therefore, the results from our qRT-PCR indicate that NF-κB signaling is involved in the regulation of RANKL and PTHrP, the osteoclastogenesis-associated genes in PCa cells.

### PCa cells capable of growing in the bone microenvironment have higher NF-κB activity

**Figure 2:**
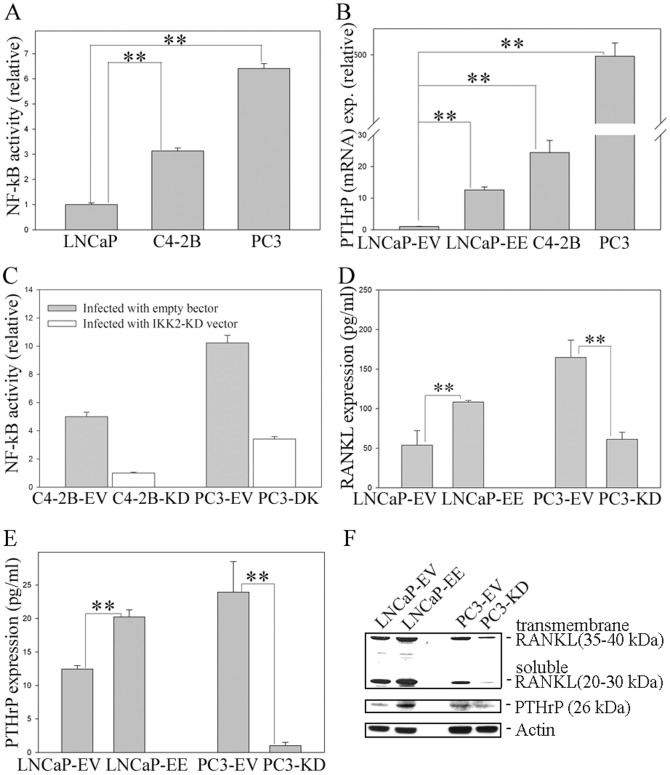
Activation of NF-κB signaling increases RANKL and PTHrP proteins expression in PCa cells. (A) PCa cells capable of growth in the bone microenvironment exhibit increased NF-κB activity. NF-κB activity in LNCaP, C4-2B and PC3 cells were measured by using a NF-κB responsive NGL reporter. (B) Expression of PTHrP is correlated with the activity of NF-κB signaling in PCa cells. Expression of PTHrP in LNCaP, NF-κB activated LNCaP-EE, C4-2B and PC3 cells was measured by qRT-PCR. (C) Generating NF-κB inactivated PCa cell lines. NF-κB signaling was inactivated in C4-2B and PC3 cells by infecting with IKK2-KD retroviral vector (C4-2B-KD and PC3-KD), while PCa cells infected with the empty vector were used as the controls (C4-2B and PC3-EV). NF-κB activity was measured by using a NF-κB responsive NGL reporter. (D and E) Expression of RANKL (D) and PTHrP (E) proteins in NF-κB activated LNCaP-EE and NF-κB inactivated PC3-KD cells were measured by ELISA and RIA assays, respectively. The cells infected with empty vector (LNCaP-EV and PC3-EV) were used as control. (F) Expression of RANKL and PTHrP are correlated with the activity of NF-κB signaling in PCa cells. RANKL and PTHrP expression were evaluated by Western blot analysis. The values plotted represent the mean of at least three individual samples ± SEM. Statistical significance was determined by two-sample *t*-test. **, *P*<0.01.

It is reported that C4-2B and PC3 PCa cells grow well, but LNCaP cells are difficult to grow in murine bone following intratibial/intrafemural injection [46]. This indicates that cell-specific gene expression events contribute to successful colonization of the bone by PCa cells. In order to determine whether NF-κB activity reflects PCa cells growth in the bone, endogenous NF-κB activity in LNCaP, C4-2B and PC3 cells was measured using the NGL reporter [35]. Interestingly, NF-κB activity in C4-2B and PC3 cells (which grow well in the bone) is significantly higher than that of LNCaP cells (**Fig. 2A**). In addition, qRT-PCR indicate PTHrP (mRNA) expression is significantly higher in NF-κB activated LNCaP-EE, C4-2B and PC3 cells, compared with that in LNCaP cells (**Fig. 2B**). ELISA and RIA assays show that the activation of NF-κB signaling increased RANKL and PTHrP (protein) secretion significantly in LNCaP-EE cells, compared to empty vector control cells (**Fig. 2C, D and E**). However, inactivation of NF-κB signaling in PC3 cells (PC3-KD) by infecting them with IKK2-KD retroviral vector in which NF-κB activity was inhibited with a kinase dead (KD) IKK2 mutant [32], [33] (**Fig. 2C**), resulted in a significant decrease in RANKL and PTHrP levels compared to empty vector control cells (PC3-EV) (**Fig. 2D and E**). Western blotting analysis further confirmed that the expression of RANKL and PTHrP was increased in NF-κB activated PCa cells (LNCaP-EE), while, decreased in NF-κB inactivated PCa cells (PC3-KD), compared to empty vector control cells (LNCaP-EV and PC3-EV) (**Fig. 2F**). These results indicate that PCa cells that are capable of growing in the bone microenvironment have higher NF-κB activity and activation of NF-κB signaling up-regulates osteoclastogenesis-associated genes in PCa cells, potentially enhancing their ability to attach and grow in the bone microenvironment.

### Activation of NF-κB signaling in PCa cells contributes to osteoclastogenesis

**Figure 3:**
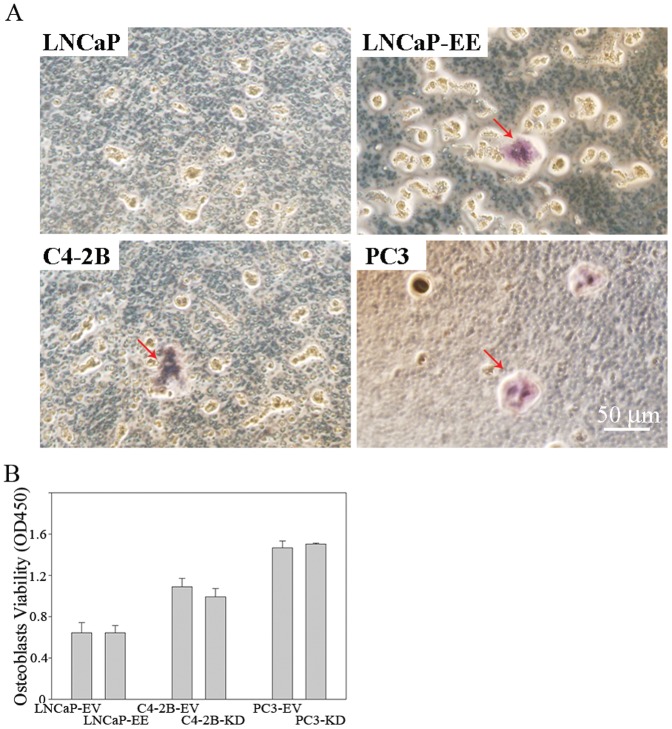
Secretions from NF-κB activated PCa cells induce osteoclast-like cell formation. (A) Conditioned medium from NF-κB activated PCa cells induces osteoclast-like cell formation *in vitro*. Bone marrow-derived macrophages were treated with conditioned media from LNCaP, NF-κB activated LNCaP-EE, C4-2B and PC3 cells. TRAP staining was performed after 10 days of additional culture with conditioned media. Arrows indicate the osteoclast-like cells. (B) Activation of NF-κB signaling in PCa cells has no significant effect on the osteoblasts proliferation. Primary cultured osteoblasts were treated with conditioned media from NF-κB activated (LNCaP-EE, C4-2B-EV and PC3-EV) or inactivated (LNCaP-EV, C4-2B-KD and PC3-KD) PCa cells. Proliferation assay (MTT assay) was performed at 48 hours after treatment.

Osteoclasts are highly specialized multinucleated cells responsible for bone resorption, a process critical for maintenance of bone structure and calcium homeostasis. However, pathological activation of osteoclasts results in bone loss associated with inflammatory arthritis, periodontal disease, and cancer metastasis to bone. Osteoclasts are derived from monocyte/macrophage lineage progenitors under the influence of the cytokine RANKL [47]. In order to determine whether activation of NF-κB signaling in PCa cells influences osteoclastogenesis in bone marrow, the monocyte/macrophage lineage progenitors from primary bone marrow cells were treated with conditioned medium from NF-κB activated/inactivated PCa cells, and monitored for osteoclast differentiation. Our results show that conditioned medium from NF-κB activated LNCaP cells (LNCaP-EE) induced TRAP-positive multinuclear cell formation characteristic of osteoclasts, while the conditioned medium from LNCaP cells failed to do so (**Fig. 3A** and **Table 1**). C4-2B and PC3 cell conditioned media also induced TRAP-positive multinucleated cell formation (**Fig. 3A** and **Table 1**). To further understand how NF-κB signaling in PCa cells influences cellular components of the bone microenviornment, osteoblasts from primary cultures of murine bone marrow cells were treated with conditioned medium from NF-κB activated/inactivated PCa cells. Our results show that conditioned medium derived from NF-κB activated PCa cells had no significant effect on osteoblast proliferation (**Fig. 3B**). These results indicate that activation of NF-κB signaling in PCa cells contributes to osteoclastogenesis, but not to osteoblast proliferation.

**Table 1:** Quantification of octeoclasts induced by conditioned medium derived from PCa cells.

Source cells of conditioned medium	Number of osteoclast (Mean±SD/well of 24-well plate)
LNCaP	0
LNCaP-EE	7.7±1.21
C4-2B	8.0±2.45
PC3	11.7±2.25

### Antagonizing NF-κB signaling inhibits PCa tumor establishment and growth in the bone

**Figure 4:**
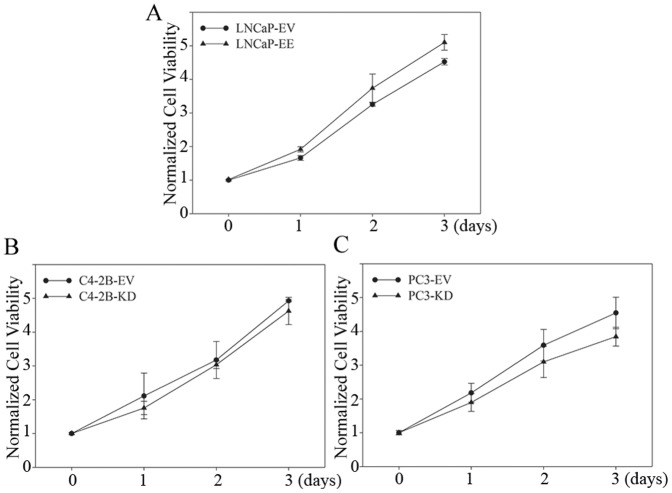
Activation of NF-κB signaling in PCa cells has no significant effect on the cell proliferation rate *in vitro*. NF-κB activated/inactivated PCa cell lines were generated by stably infecting with IKK2-EE/IKK2-KD vectors. IKK2-EV was used as control vector. Proliferation rate was determined by MTT assay.

**Figure 5:**
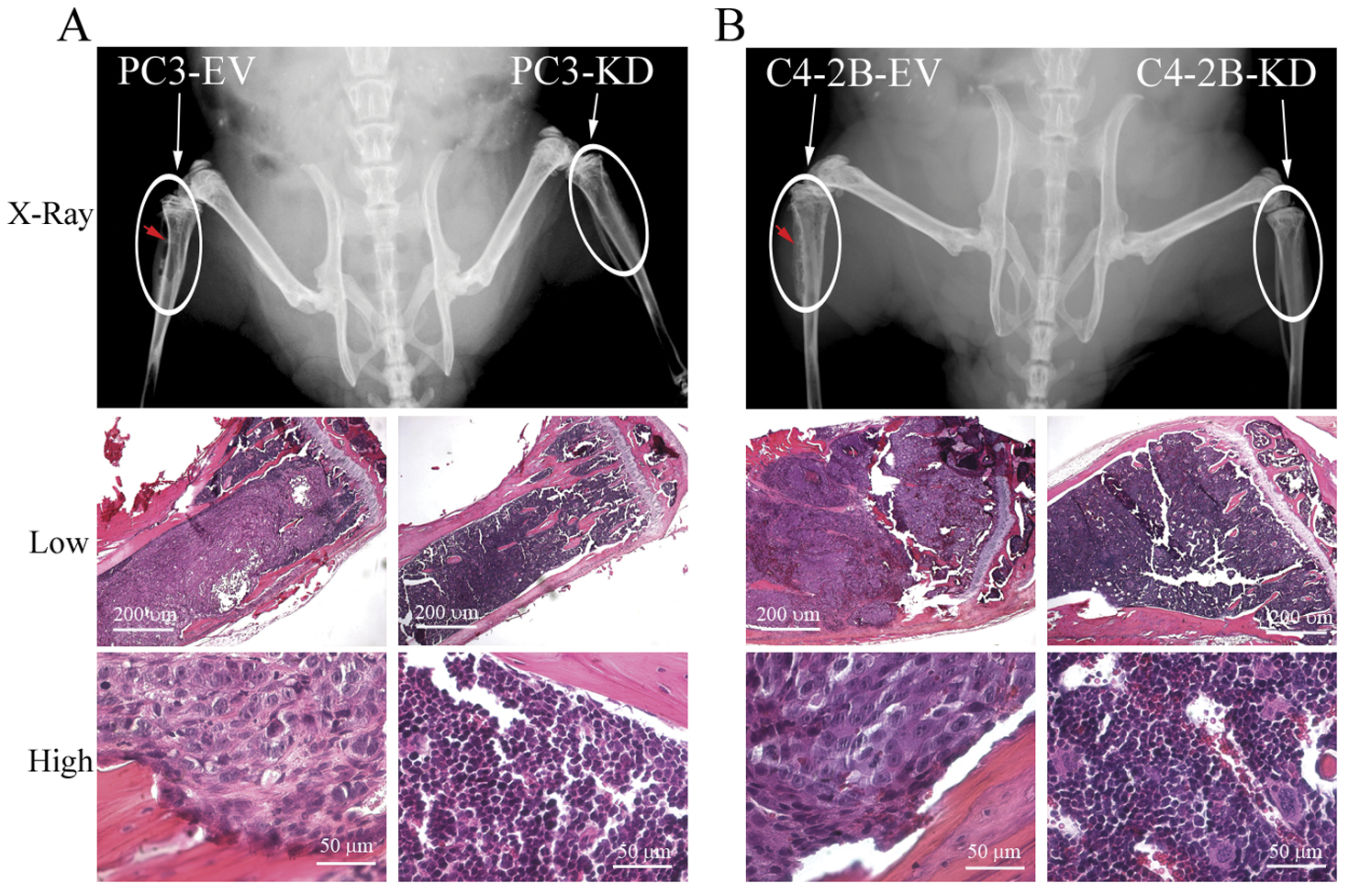
Inactivation of NF-κB signaling in PCa cells inhibits tumor establishment and growth in the bone microenvironment. NF-κB inactivated PC3-KD and C4-2B-KD cells were grafted into the mouse bones by intratibial injection. PC3 and C4-2B cells infected with empty vector (PC3-EV and C4-2B-EV) were used as controls. (A) Tumor formation of PC3-EV and PC3-KD was determined by small animal X-ray radiograph imaging and histological analysis at 3 weeks after the grafting. (B) Tumor formation of C4-2B-EV and C4-2B-KD was determined by small animal X-ray radiograph imaging and histological analysis at 10 weeks after the grafting. Arrows indicate the sites of tumor formation in the bone.

In order to determine if antagonizing NF-κB signaling inhibits PCa tumor establishment and growth in the bone microenvironment, we generated NF-κB signaling inactivated PCa cell lines by stably infecting with IKK2-KD vectors (PC3-KD and C4-2B-KD) (**Fig. 2C**). Although NF-κB blockade slightly altered proliferation rates (**Fig. 4**), all of the engineered cells grew well (survive) *in vitro* after down regulation of NF-κB activity. NF-κB inactivated PC3 and C4-2B cells (PC3-KD and C4-2B-KD) were inoculated into the bone of mice by intratibial injection, and small animal X-ray radiograph imaging was performed to monitor bone lesion development. Mice were sacrificed for histological analysis at 3 weeks (for PC3-EV and PC3-KD cells injected mice) and 10 weeks (for C4-2B-EV and C4-2B-KD cells injected mice) post-inoculation. All tumors grafted with PC3-EV and C4-2B-EV cells (PC3 and C4-2B cells infected with empty vector, were used as controls) grew well in the bone; whereas, NF-κB inactivated PC3-KD and C4-2B-KD cell growth was not detected (0/4 in both group) by X-ray radiograph imaging or histological analysis (**Fig. 5A and 5B**). Histomorphometric analyses showed that the average BV/TV is 5.3% for PC3-EV and 13.9% for C4-2B-EV tumors. These results indicate that inactivation of NF-κB signaling inhibits both osteolytic (PC3) and osteoblastic/osteoclastic mixed (C4-2B) PCa tumors establishment and growth in the bone environment.

### Activation of the NF-κB signaling contributes PCa tumor establishment and growth in the bone

**Figure 6:**
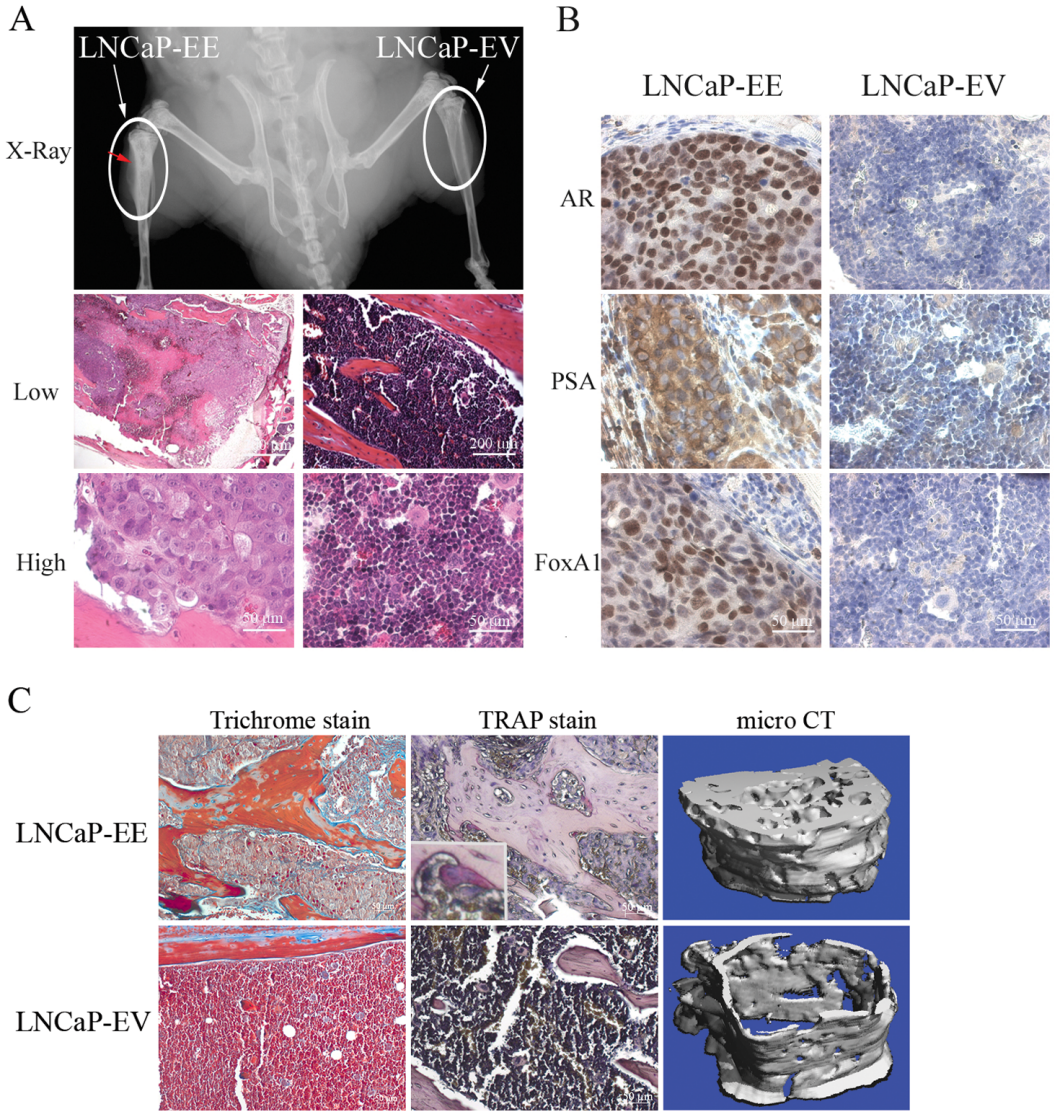
Activation of NF-κB signaling in PCa cells contributes to tumor establishment and growth in the bone microenvironment. NF-κB activated LNCaP-EE cells were grafted into the mouse bones by intratibial injection. LNCaP cells infected with empty vector (LNCaP-EV) were used as controls. (A) NF-κB activated LNCaP-EE cells growth in the bone was detected by small animal X-ray radiograph imaging and histological analysis (H&E staining) at 10 weeks after the grafting. (B) Immunohistochemical staining of AR, PSA and FoxA1 was performed to confirm that the cells growing in the bone are human PCa cells. Arrow indicates tumor formation in the bone. (C) Trichrome blue and TRAP staining were performed to confirm osteoblastic lesions and osteoclasts differentiation surrounding the new formed bone. Micro CT scanning showed osteoblastic lesions in the bone when injected with LNCaP-EE cells.

In order to further confirm the role of NF-κB signaling in PCa bone metastasis, we grafted NF-κB activated LNCaP cells (LNCaP-EE) (**Fig. 1A**) into the mouse bone by intratibial injection. No growth (0/4) was detected by either X-ray radiographic imaging or histological analysis for control LNCaP-EV cells (LNCaP-EV: infected with the empty vector) grafted into the bone after 10 weeks. Importantly, the NF-κB activated LNCaP-EE cells grew well (3/4) in the bone as detected by X-ray radiograph imaging (**Fig. 6A**). These results were confirmed by H&E and IHC staining using antibodies of prostate specific antigen (PSA) and FOXA1 (marker of prostate epithelium) (**Fig. 6A and 6B**). Histomorphometric analyses showed that the average BV/TV is 31.2%. Further, trichrome blue staining and micro CT scanning also showed that NF-κB activated LNCaP-EE cells induce osteoblastic/osteoclastic mixed lesions in the bone (**Fig. 6C**). TRAP staining shows that many TRAP-positive osteoclasts exist surrounding the new formed bone (**Fig. 6C**). These results suggest that activation of NF-κB signaling in PCa cells increases osteoclast differentiation which provides a sufficient microenvironment to enable tumor cell survival, growth and pathological bone remodeling in osseous PCa metastases.

## Discussion

PCa metastasizes to bone in a non-random manner, and numerous pieces of evidence support the concept that tumor colonization of the bone is an active process involving reciprocal stimulation between PCa cells and cellular elements in the bone matrix [48], [49]. After PCa cells reach the bone, the interaction between the tumor cells and resident cells within the bone marrow, are important for creating a ‘fertile’ environment to support the ability of metastatic PCa cells to escape apoptosis and to optimize their survival [50], [51]. It is established that up-regulation of osteoclastogenesis in the bone microenvironment is a necessary prerequisite for the ability of cancer cells to successfully colonize bone both in osteolytic and osteoblastic bone metastasis [1], [4]. Although several studies indicate that many factors secreted from PCa cells regulate osteoclastogenesis and strongly suggest an important role for osteoclast function in the successful formation of PCa bone metastases, the identification of the primary pathway that regulates the expression of osteoclastogenic gene(s) that contribute to the ability of PCa cells to attach and grow in the bone environment remains unclear. In this study, we demonstrated that PCa cells, which grow in the bone microenvironment, have higher levels of NF-κB activity (**Fig. 2A**). In addition, activation of the NF-κB signaling increases osteoclastogenic genes (RANKL and PTHrP) expression in PCa cells (**Fig. 1** **,** **2**) and contributes to osteoclastogenesis in the bone environment (**Fig. 3**). RANKL and PTHrP are known as key factors of osteoclastogenesis in the bone, and they play a key role in the bone metastasis of many cancers [15]. Therefore, increased expression of osteoclastogenesis-associated genes, such as RANKL and PTHrP, in response to activation of NF-κB signaling in PCa cells could be important contributors to PCa cell establishment in the bone, perhaps by osteolytic effects.

It is known that the PC3 cell line exhibits osteolytic activity, but C4-2B is an osteoblastic/osteoclastic mixed PCa cell line [46]. Our studies demonstrate that activation of NF-κB signaling contributes to osteoclastogenesis in the bone environment (**Fig. 3**) and inactivation of NF-κB signaling inhibits tumor growth in the bone in both of PC3 and C4-2B cells (**Fig. 5**). These results indicate that an osteoclastogenic response to activation of NF-κB signaling in PCa cells may be sufficient for both osteolytic and osteoblastic bone metastasis of PCa, and the activation of NF-κB signaling contributes to bone metastasis in both of the osteolytic and osteoblastic PCa cells.

Besides increasing RANKL expression in the osteoblast, PTHrP also regulates osteoblast proliferation and differentiation in a temporal and dose dependent manner [52], [53]. Bone turnover has been implicated in the localization of tumors to bone and PTHrP increases bone turnover. Bone turnover results in the release of growth factors such as TGFβ and minerals such as calcium, both of which impact tumor cell growth and contribute to continued PTHrP production. PTHrP also has anabolic properties and could be in part responsible for osteoblastic type reactions in PCa [15]. However, our studies show that although activation of NF-κB signaling in PCa cells increased PTHrP expression significantly, while it has no significant effect on the osteoblasts proliferation *in vitro* (**Fig. 3B**). Therefore, increased PTHrP expression induced by activation of NF-κB signaling in PCa cells may contribute to induce osteoclasts differentiation thereby provide sufficient microenvironmental cues (i.e., increased solubility of bone matrix-associated growth factors) to promote PCa cells attachment and growth in the bone. However, whether activation of NF-κB signaling in PCa cells may contribute to ostoeblast differentiation needs to be further studied.

LNCaP is known as an osteoblastic PCa cell line [46] and has a low level of NF-κB activity compared with other PCa cells (**Fig. 2A**). LNCaP cells do not grow well in the bone even though they express moderate levels of osteolytic factors, such as RANKL and PTHrP (**Fig. 2**). Although activation of NF-κB signaling in PCa cells contributes to osteoclastogenesis *in vitro* by regulating osteoclastogenic genes (**Fig. 1** **,** **2** and **3**), when NF-κB was activated in the LNCaP cells in the bone microenvironment, they formed typical osteoblastic/osteoclastic mixed lesions (**Fig. 6**). We do not know which signal(s) or pathway(s) in LNCaP cells that predispose this widely used cell line to osteoblastic growth. Based upon our studies, NF-κB signaling is required for successful growth of this particular PCa cell in the bone microenvironment (as evidenced by the ability to promote LNCaP growth in the bone by activating the NF-κB pathway). This would indicate that activation of NF-κB in LNCaP followed by intratibial injection may result in an initial rapid lytic response, which occurs before the development of overt (and detectable) metastases. Over time, after the initial lytic phase passes, LNCaP cells may begin to assume an osteoblastic phenotype, and subsequently detectible metastatic lesions. This is supported by our observation that treatment of bone marrow progenitor cells with conditioned medium derived from NF-κB activated LNCaP-EE cells resulted in increased TRAP staining (**Fig. 3**), and although LNCaP-EE cells formed osteoblastic lesions in the bone, TRAP staining shows that many TRAP-positive osteoclasts exist surrounding the new formed bone (**Fig. 6C**). This is also supported by the observation that the lineage-related, castrate resistant C4-2B cell line, which itself was derived from LNCaP passage through a castrate host, exhibits increased NF-κB signaling and exhibits an osteoblastic/osteoclastic mixed phenotype. Another possibility is that simultaneous activation of multiple pathways (in addition to NF-κB) is responsible for the net osteoblastic influence of PCa cells within the bone microenvironment.

A role for the NF-κB pathway in PCa metastasis has been previously described [18], [19], [28], [29]. However, these studies were focused on the role of in the formation of visceral or “soft tissue” metastasis to the lymph node and liver. There indeed exists strong evidence of a role for increased NF-κB pathway activity in the ability of another osteotropic malignancy, namely breast cancer, to form boney metastasis [54], [55]. A major limitation in the identification of the molecular mechanisms responsible for PCa bone metastasis is the extreme difficulty in obtaining human specimens from patients with boney metastasis. While this is an obstacle faced by the whole PCa research community, the situation is improving with the advent of specialized tissue procurement programs. To the best of our knowledge, this is the first report of a role for NF-κB pathway in PCa bone metastasis, and future studies should be directed at determining the extent to which increased NF-κB pathway activity contributes to human PCa bone metastasis. This should be a priority as inhibition of the NF-κB pathway could provide a novel therapeutic approach for disease management.

**Figure 7:**
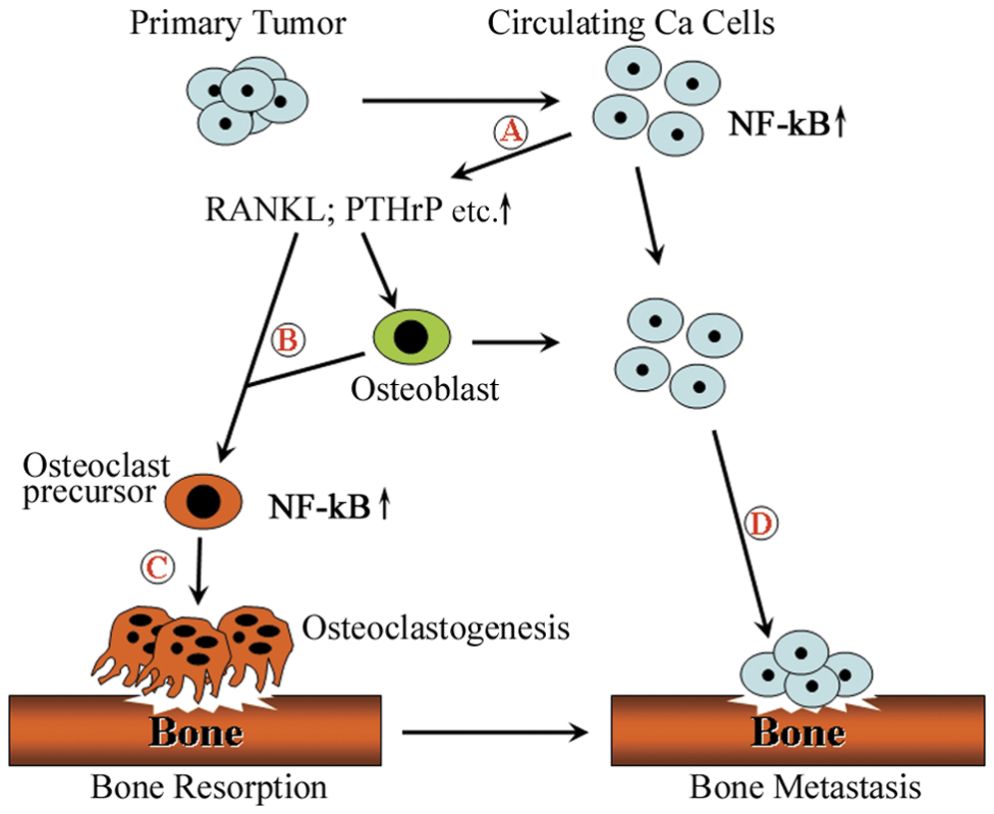
Schematic representation of the role of NF-κB signaling in the establishment of PCa bone metastases. (A) The activation of NF-κB signaling in PCa cells increases the expression of osteoclastogenesis-associated genes (such as RANKL, PTHrP etc.). (B) These elevated osteolytic factors act as paracrine growth factors to affect directly/indirectly the osteoclast precursor to promote osteoclastogenesis in the bone microenvironment. (C) Activated osteoclastogenesis will induce osteoclastic bone resorption. (D) Therefore, osteoclastic bone resorption induced by the osteolytic factors from PCa cells is sufficient for cancer cells attachment and the establishment of tumors in the skeleton.

In summary, the results of our study indicate that active NF-κB signaling in PCa cells promotes expression of osteoclast differentiation associated genes (such as RANKL, PTHrP etc.), thereby promoting tumor-induced bone disease. We have developed a working model that integrates our findings regarding PCa growth in the bone microenvironment (**Fig. 7**). Activation of NF-κB signaling elevates the expression of osteolytic factors (such as RANKL, PTHrP etc.) in PCa cells. The increased levels of these factors will result in either direct or indirect effects on osteoclast differentiation to promote osteoclastogenesis and bone resorption in the bone microenvironment. For example, RANKL responds to NF-κB signaling in the osteoclast by directly binding to RANK, its receptor, to stimulate osteoclast differentiation; while PTHrP up regulates the RANKL in osteoblasts. The increased RANKL will further promote osteoclastogenesis by promoting osteoclast differentiation. Thus, osteoclastic bone resorption caused by the osteolytic factors derived from cancer cells will provide sufficient microenvironment for cancer cells survival and growth in both of osteolytic and osteoblastic cancer, thereby contributes to cancer cell colonization and the establishment of tumors in the skeleton.
